# Securing Rare Earth Permanent Magnet Needs for Sustainable Energy Initiatives

**DOI:** 10.3390/ma17225442

**Published:** 2024-11-07

**Authors:** Dan-Cristian Popa, Loránd Szabó

**Affiliations:** Electrical Machines and Drives Department, Technical University of Cluj-Napoca, 400114 Cluj-Napoca, Romania; dan.cristian.popa@emd.utcluj.ro

**Keywords:** generators, machinery production industries, permanent magnet machines, permanent magnets, rare earth metals, supply chain management

## Abstract

Rare earth permanent magnets are vital in various sectors, including renewable energy conversion, where they are widely used in permanent magnet generators. However, the global supply and availability of these materials present significant risks, and their mining and processing have raised serious environmental concerns. This paper reviews the necessary legislative, economic, and technological measures that must be implemented to address these issues. While it may not be feasible to eliminate the risks associated with the availability of rare earth materials, researchers in the field of electrical generators can play a crucial role in significantly reducing the demand for newly mined and processed such materials, thereby mitigating the negative environmental impacts of their extraction and production.

## 1. Introduction

For achieving the sustainable development of society, it is crucial to mitigate the detrimental environmental effects caused by intensive urbanization, industrialization, and resource exploration. This can only be accomplished by pursuing a green transition, which involves moving towards an economy that does not depend on fossil fuels or the overuse of natural resources [1].

To accomplish this vital objective for humanity, several key policies can be implemented [2]. These efforts include promoting decarbonization by means of the use of renewable energy sources, advancing green transportation and manufacturing, transitioning to a circular economy, increasing energy efficiency, and implementing additional sustainability measures [3,4].

Governments of all developed countries endeavor to achieve these objectives and strive to harmonize their legal structure with these aspirations to safeguard and rejuvenate the environment. Recently introduced regulations outline specific goals, performance indicators, and limitations aimed at promoting and advancing this important initiative. A clear example is the launch of the “European Green New 27” strategy by the European Union (EU). This is designed to promote the adoption of renewable energy sources and electric vehicles. Additionally, the EU has introduced the “Fit for 55—The EU’s Plan for a Green Transition” package, which aims to align its policies to achieve climate neutrality by 2050 and reduce greenhouse gas emissions by at least 55% compared to 1990 levels by 2030 [5,6]. Similarly, highly industrialized countries like the U.S. and China also have implemented ambitious regulations and policies in line with these objectives [7,8].

The considerations presented above highlight the critical role of renewable energy technologies in achieving sustainable development objectives, emphasizing the need for their intensive and efficient utilization. Wind, along with the sun, possesses immense potential as an energy resource [9]. The conversion of these resources is experiencing significant and continuous growth, making them valuable contributors to the sustainable development goals of numerous countries, including those that are yet underdeveloped. According to forecasts, it is anticipated that the worldwide installation of wind turbines (WT) will reach a cumulative capacity of 680 GW for the next five years [10].

Permanent magnet (PM) electrical machines (EMs) operating in generator mode play a significant role in renewable energy systems. They are widely used in devices that harness wind, wave, and tidal energy, as well as in smaller household electric generators (EGs) [11,12,13].

Rare earth materials (REMs) are essential for the manufacturing of powerful PMs used in these devices. Approximately 35% of the REMs processed globally are utilized in the creation of these hard magnetic alloys, which are mainly used in EMs [14]. Therefore, it can be concluded that renewable energy conversion, so much needed for the sustainable development of human society, strongly depends on these very particular REMs.

Unfortunately, numerous political, economic, and environmental concerns about the supply of REMs pose a significant threat to their accessibility. It is imperative to address these risks to ensure their availability.

This paper aims to examine the primary hazards associated with the availability of REMs required for PMs, as well as explore potential solutions within the field of EMs to mitigate them. The initial part of the article emphasizes the necessity of REMs in renewable energy conversion and provides an in-depth analysis of the risks associated with their availability and the unpredictability of their prices. An entire section surveys the principal methods of mitigating the supply hazards of these crucial materials through legislative, economic, and technological measures. The most consistent last part of the article deals with various EM topologies that have the potential to reduce dependence on REMs.

## 2. Need for Rare Earth Materials in Sustainable Energy Conversion

The group of metals known as “rare earths” consists of 17 elements located in the central section of the periodic table, with atomic numbers 21, 39, and 57 through 71. A great share of the REMs produced, accounting for 20%, are utilized in the manufacturing of PMs [14]. Such materials are neodymium, samarium, dysprosium, praseodymium, terbium, and gadolinium [15,16].

NdFeB alloys are the most commonly used PMs based on REMs. Among all types of PMs, they have the highest magnetic energy (BH_max_) of 430 kJ/m^3^. Usually, they contain a great amount of iron, around 35% neodymium, less than 10% dysprosium, and smaller amounts of gadolinium, praseodymium, and terbium [17]. The last materials are utilized as additives to enhance coercivity and temperature stability. These PMs may have a maximum remanence (B_r_) of 1.49 T and coercivity (H_c_) up to 1.2 MA/m.

SmCo PMs are typically composed of approximately 35% samarium and 60% cobalt, along with small amounts of iron, copper, zirconium, hafnium, and praseodymium. Their typical variants may have 250 kJ/m^3^ maximum energy [18].

**Figure 1:**
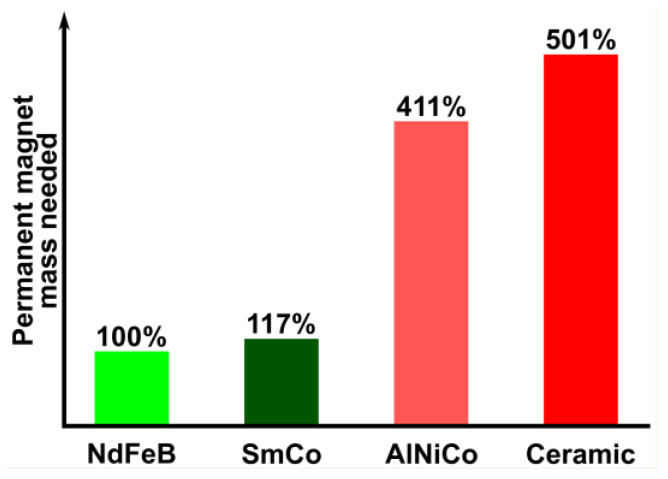
The relative masses of the available PMs needed to produce an identical magnetic force.

These two types of rare earth (RE) PMs are recognized as the best PMs due to their significantly higher specific energy compared to other available types such as ceramic (ferrite) or AlNiCo PMs. This results in a reduced mass of RE PMs required to generate the same magnetic force, as depicted in Figure 1. As a result, the use of these PMs significantly reduces the volume and weight of the equipment into which they are incorporated.

**Figure 2:**
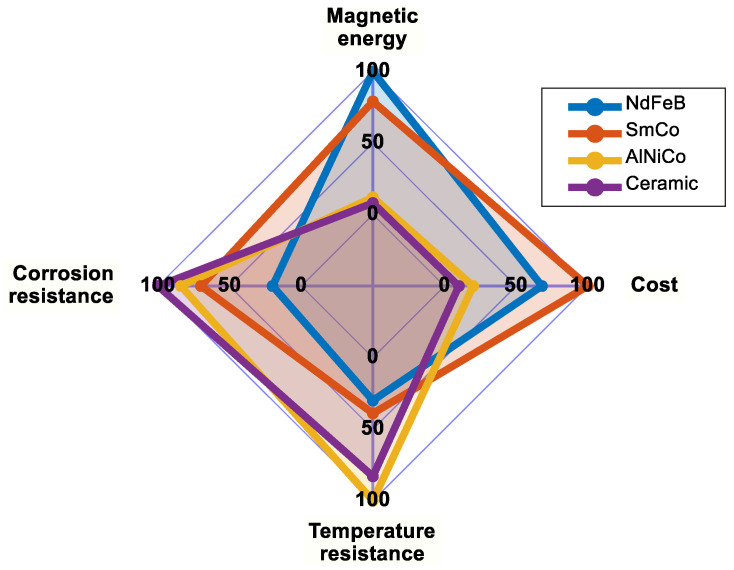
Comparison of the main characteristics of common PMs (0 = poor, 25 = fair, 50 = good, 75 = very good, and 100 = excellent).

However, RE PMs have several common drawbacks. REMs are not only scarce but also costly. NdFeB PMs are particularly vulnerable to oxidation and corrosion, which occurs when they are exposed to moisture, leading to rust and gradual degradation over time. Additionally, these PMs exhibit significant temperature sensitivity, beginning to lose their magnetic properties at temperatures below the usual maximum permissible limits for EMs (115 °C in F-class insulation systems). In contrast, RE-free PMs do not require anti-corrosive coating and possess very low density along with excellent mechanical properties [19]. Figure 2 presents a spider plot illustrating the comparison of key properties among the main types of PMs. This visualization highlights each PM type’s performance across essential characteristics for clearer comparison.

EMs are among the primary users of RE PMs due to the numerous advantages offered by permanent excitation assured by them. Since they do not require electrical energy to generate the field, there are no excitation losses, resulting in higher efficiency. Additionally, the use of smaller quantities of RE-based PMs allows for reduced iron core and winding sizes, resulting in higher power and torque density. In comparison to electromagnetically excited EMs, those with PMs typically possess lighter rotor masses, thereby exhibiting superior dynamic performances [20].

Synchronous machines are the most widely utilized EMs that incorporate RE PMs. These also find extensive applications in renewable energy conversion, particularly in WTs [21]. In the context of high-power EGs used in WTs, two primary technological approaches are employed: gearbox-based and direct-driven systems. Gearboxes enable the adjustment of the turbine’s slow speed to the higher speed required by the double-fed induction generators. However, these gearboxes introduce additional losses and noise. Alternatively, synchronous generators with numerous pole pairs having low values of the rated speed can be directly connected to the WT’s shaft. These generators necessitate intense magnetic fields, which can be efficiently achieved through the use of PMs [22].

The continuous increase in WT power capacities, hub heights, and rotor diameters necessitates the use of EGs with higher power ratings. These demand substantial amounts of high-quality PMs, with a minimum requirement of 600 kg/MW. The forecasts indicate that within the next five years, 204 kt of PMs will be needed solely for these EGs [23].

REMs play a crucial role in various other essential components of emerging power systems. They are used as components in various parts of next-generation solid oxide fuel cells, including the cathode, anode, solid electrolyte, and diverse connectors [24,25]. These materials are also employed as advanced electrode materials, which have the potential to improve the power density, charge and discharge time, and cycle life of supercapacitors used in energy storage systems [26]. Furthermore, REMs are extensively being investigated in the field of hydrogen energy devices, encompassing both the generation and storage aspects [25].

Numerous ongoing research activities are currently dedicated to improving solar panels by the use of REMs [27,28]. Advanced solar panels possess the capability to enhance photon absorption across a wide range of wavelengths, extend the lifespan of photoelectrons, and reduce charge recombination. Moreover, with the utilization of REMs, the efficiency of antireflection and self-cleaning coatings can be optimized [25,29].

Intensive developments are also being pursued in the field of superconducting EGs and electrical energy storage systems. These research activities also involve the exploration of REMs [30]. Various compounds, such as the RE–barium–copper oxide (ReBCO), demonstrate high-temperature superconductivity, meaning they can conduct electricity without any resistance even at relatively higher temperatures. For such special superconducting materials, researchers are investigating REMs such as yttrium, lanthanum, samarium, and neodymium [31].

In most applications, apart from PMs, a relatively small quantity of REM is common, typically ranging from 0.1% to 5% by weight [32]. Despite their limited quantity, they significantly contribute to improving the quality and performance of the aforementioned sustainable energy conversion-related applications.

The demand for REMs, particularly in the production of high-quality PMs, is substantial and rapidly increasing, as evidenced by the presented aspects. As a result, their availability on a global scale is of utmost importance for numerous critical industries in all industrialized countries.

## 3. Risks of Availability and Price Unpredictability of the Rare Earth Materials

The surge in demand for REMs in the foreseeable future is anticipated to exhibit radical growth. Specifically, the utilization of PMs only in wind energy conversion EGs will necessitate significant quantities by 2040, with a remarkable escalation of 31, 20, 16, and 13 times the current demand for praseodymium, terbium, neodymium, and dysprosium, respectively [33]. However, this escalating demand gives rise to various apprehensions regarding their general availability.

To understand these strategic vulnerabilities, the main supply chain of these materials must be considered [34]. The focus must be set on the mining and processing of the REMs and manufacturing PMs.

**Figure 3:**
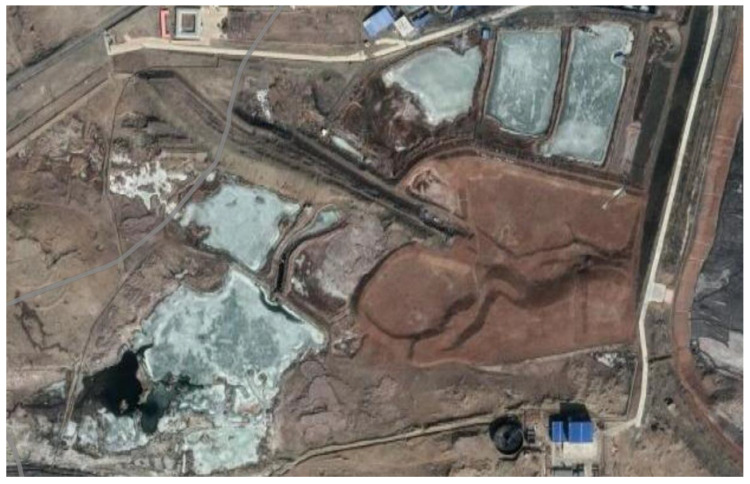
Satellite view of the Bayan Obo district (the world’s biggest REM mine), Baotou (Inner Mongolia, China) [36].

RE ore mining is a hard, time-consuming, and energy-consuming process. There are two distinct methods for this purpose. Upon the first one, large holes are drilled into the ground, followed by the insertion of plastic pipes. Subsequently, appropriate chemicals are injected into the soil to dissolve the REMs, which are then extracted from deep beneath the surface. The second method involves the excavation of large open pits by removing the topsoil to access the desired minerals. In both cases, a significant quantity of toxic solid waste or leachate remains after the mining process, as can be seen in Figure 3. All of these are posing a perpetual environmental threat [35].

The mining of REMs also presents other significant environmental and health-related challenges. The use of chemicals in the extraction process contaminates local water sources. Furthermore, the dust and gases released during mining and processing contribute significantly to air pollution, adversely affecting local air quality and increasing greenhouse gas emissions [37]. Mine workers and the surrounding communities face exposure to hazardous substances used in the mining process, which can lead to skin irritation, respiratory issues, and other serious health problems. Additionally, radioactive elements found in RE ores, such as thorium and uranium, pose serious health risks if not properly managed. Prolonged exposure to these elements may significantly elevate the risk of cancer and other life-threatening health conditions [38]. The mining operations often involve heavy machinery and hazardous working conditions, resulting in a higher incidence of accidents and injuries among workers [39].

Mined RE ores are known for their mineralogical and chemical intricacy. Extracting REMs from these ores is challenging, as they often contain multiple valuable minerals, each requiring separate extraction, purification, and processing techniques. These pose a significant technological challenge, and inherently a cost-related risk [40].

All developed countries have demonstrated a heightened dedication to actively participating in global endeavors aimed at addressing the challenges of climate change. As a result, stricter mining and material processing regulations that prioritize the protection of the environment are being implemented, leading to a definitive rise in costs and an indirect adverse effect on the pricing of REMs.

**Figure 4:**
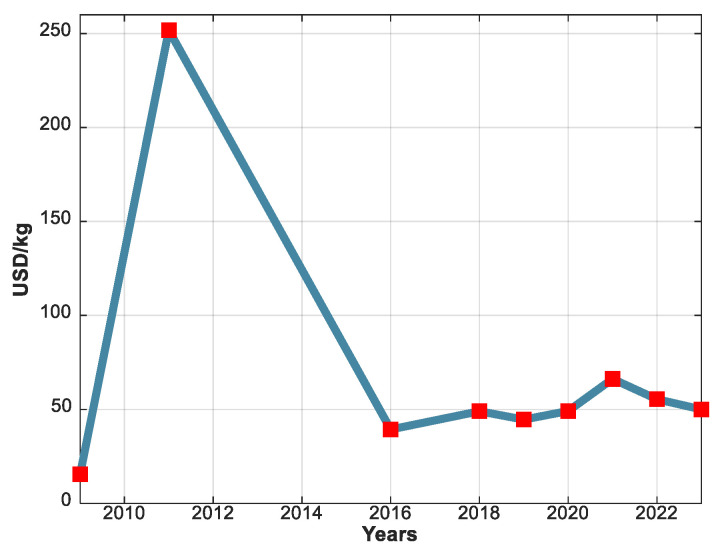
Neodymium oxide price evolution from 2009 until today.

China is the world leader in the production, extraction, and refining of REMs since it owns 33.85% of the worldwide reserves [41]. Their monopoly over these crucial materials is very dangerous for other developed countries since it created and will also certainly result in future serious geopolitical and economic uncertainties, which essentially depend on the internal politics and economy of this country and its relations with others [42,43]. China used the REMs market as an economic trap for many years. The first embargo occurred in 2010, resulting in a 37% reduction in the country’s REM exports and an unprecedented price surge. A year before, the cost of neodymium oxide (the fundamental raw material for NdFeB PMs) was only 15.4 USD/kg (see Figure 4). However, by 2011, this price had increased to over 250 USD/kg, representing a surge of more than 16 times. Although the prices of REMs subsequently declined to approximately 50 USD/kg (with the current price as of 2022 being 55.4 USD/kg), they never reverted to the low levels observed before the 2011 crisis [44,45].

Currently, the supply chains of REMs are facing extra major obstacles. Despite the gradual return to normalcy following border closures and the decrease in manufacturing and transportation activities due to the COVID-19 pandemic, the supply chains have not fully recuperated. It is expected that this pandemic will continue to have a prolonged impact on the global economy [46,47]. The ongoing conflict between Russia and Ukraine has further disrupted supply chains, primarily due to the implementation of economic sanctions against Russia [48]. Moreover, starting in early 2024, global freight transportation faced significant disruptions at two critical shipping routes: the Suez Canal, due to attacks on vessels in the Red Sea area, and the Panama Canal, due to severe droughts. These two canals typically handle 15% and 5% of global maritime trade volume, respectively. The resulting delays, caused by using longer shipping routes, have severely impacted the supply chains of REMs, further exacerbating the situation [49].

As a result of the growing demand for REMs in various strategic industries, including electric power production, and the potential risks associated with their limited supply, these resources are categorized as “critical minerals” or “critical metals” [50]. Consequently, numerous steps have been implemented in developed countries to alleviate the aforementioned hazards.

## 4. Mitigating the Supply Risks of Rare Earth Materials

To mitigate the risks of availability and price unpredictability associated with REMs, three approaches must be mandatorily implemented [51]:

1. Imposing legislative and economic measures;
2. Employing more sustainable material management methods;
3. Reducing REM consumption [52].

### 4.1. Legislative and Economic Measures

To alleviate the risks associated with the supply of critical materials, industrialized countries have implemented various legislative measures [51]. These aim to promote the local mining and processing of REMs through centralized policies, strategic investments, tax reductions, and other financial assistance [53,54]. It is expected that these measures will contribute to the increase in the domestic manufacturing of RE-based PMs. Emphasis is placed also on fostering collaboration among developed countries to reduce their reliance on China for critical minerals.

#### 4.1.1. Reopening Mines While Upholding Stringent Environmental Regulations

Legislative measures are not sufficient to achieve the desired aims. Consequently, economic and technical actions have also been initiated to facilitate local extraction, processing, and production [51]. As a result, RE ore mines like the Californian Mountain Pass Mine (U.S.) and the Norra Kärr in Sweden have been reopened. Additionally, new deposits have been discovered in several places around the world—in Australia, Brazil, Europe, etc. Both the U.S. and Europe are currently more and more involved in the separation and refinement of REMs, with a particular emphasis on transforming them into end-products like PMs [55,56,57].

It is imperative to consider the environmental, health, and social implications associated with mining activities [37]. In the case of the two reopened mines mentioned earlier, stringent environmental regulations are taken very seriously to ensure sustainable and responsible mining practices. Future mining operations at these locations must comply with rigorous standards, including U.S. federal and California state environmental regulations, which are among the most stringent in the world [58]. The Swedish mining company was required to conduct a Natura 2000 assessment when applying for a mining concession, ensuring that potential impacts on protected areas and biodiversity are thoroughly evaluated [59]. At both mining sites, the dry tailings process is employed to minimize the risk of groundwater contamination. This method involves storing dry tailings in lined impoundments, significantly reducing environmental risks [60]. Special attention is given also to water management. In the U.S. mine, a closed-loop, zero-discharge system for water management is implemented, meaning that all process water is recycled on-site with no discharge of wastewater into the environment. Meanwhile, at the Norra Kärr mine, the revised project design predicts a 51% reduction in water requirements over the mine’s lifespan compared to previous plans. This is achieved by utilizing mine dewatering for processing, which nearly eliminates the need for supplementary water. Additionally, chemical processing has been moved off-site, further minimizing the environmental impact [60].

All these measures collectively aim to significantly reduce the environmental footprint by minimizing the risk of chemical contamination and ensuring that these mines operate in an environmentally responsible manner, contributing to a more sustainable supply chain for REMs. These strategies should be implemented across all REM mines, not only to protect the environment but also to ensure the long-term sustainability of the industry [61,62]. However, the feasibility of this green mining approach hinges on the willingness of consumers and manufacturers to bear the higher costs associated with this method, given its inherently greater expenses [63].

#### 4.1.2. International Collaborations

The limited availability of critical materials, including REMs, has become a central focus in industrial development across leading economies [64,65]. Addressing supply limitations requires robust international collaboration to stabilize REM resources and support sustainable development across industries. Several strategies are available to achieve this.

International cooperation can help diversify supply chains, reducing reliance on any single source, a crucial factor in the REM industry. This includes developing new mining projects and investing in alternative resources across different regions [66]. In 2019, the U.S. and Australia established one of the first partnerships in this domain. Soon after, China and ASEAN (Association of Southeast Asian Nations) member countries, along with Australia, Japan, South Korea, and New Zealand, signed the Regional Comprehensive Economic Partnership (RCEP) to underscore the need for collaborative efforts that include China. In 2021, the EU and Canada launched the Strategic Partnership on Raw Materials, which promotes joint value chains, science and technology innovation, and environmental, social, and governance standards [67,68]. More recently, Japan and India partnered to leverage India’s REM resources and Japan’s technological expertise, advancing shared goals in environmental protection and technology [69].

Research and development collaborations are essential for advancing REM extraction, processing, and recycling technologies. The German Collaborative Research Center “4f for Future”, led by the Karlsruhe Institute of Technology, involves multiple European institutions focusing on REM-related technological solutions [70]. In the U.S., the Department of Energy (DOE) sponsors several initiatives, including the Critical Materials Innovation Hub and the Critical Minerals and Materials Program of the National Energy Technology Laboratory (NETL), which involve national laboratories, academia, and industry working together to build resilient REM supply chains for clean energy technologies [71,72].

Countries can also improve supply security by establishing strategic REM reserves, like oil reserves, as buffers against supply disruptions. The coordinated management of these reserves is essential, particularly with promising REM sources in deep-sea deposits [73]. Both the U.S. and Japan have initiated efforts to create such strategic REM reserves [74].

International standards can foster sustainable REM mining practices that minimize health and environmental risks [75]. The International Organization for Standardization (ISO) Technical Committee 298 and the European Committee for Standardization (CEN) Technical Committee 472 have developed standards to ensure safe and sustainable practices in REM mining, extraction, and processing [76].

Finally, trade agreements are vital for stabilizing REM supply chains by minimizing geopolitical risks [77]. Agreements like the U.S.–Mexico–Canada Agreement (USMCA) include provisions on critical minerals cooperation, bolstering North American supply chains [78]. Other notable agreements include the EU–Japan Strategic Partnership, the Australia–India–Japan Supply Chain Resilience Initiative, and various bilateral agreements between the U.S., EU, and Japan [79].

Nevertheless, it is crucial to acknowledge that solely relying on these measures will not suffice to meet the rapidly increasing industrial demands. Hence, it is vital to adopt supplementary approaches to ensure the availability of these critical materials.

### 4.2. Minimization of Mining and Utilization of Rare Earth Elements

To protect the REM deposits and preserve the environment, it is necessary to implement more effective sustainable material management practices that can aid in reducing the demand for newly extracted and refined resources. To achieve this goal for REMs, a circular economy approach must be adopted, along with enhancements in processing techniques and the reduction of their use [80].

#### 4.2.1. Applying the Circular Economy Approach

The circular economy approach emphasizes the importance of using REMs for extended periods, enabling their extraction and reuse from waste. This approach leads to the repeated utilization of materials that generate economic surplus value, reduce energy consumption, and minimize pollution. The ultimate goal is to achieve a carbon-neutral, environmentally friendly, and sustainable circular economy in the forthcoming years [81].

The possible quantity of recycled REMs from PMs is immense, as it is directly proportional to the amount of these hard magnetic materials being used [82]. Unfortunately, the current global recycling rate for REMs utilized in EMs is extremely low, standing at a mere 3%. This is alarming considering that these hard magnetic materials make up approximately 40–60% of the capital costs in PM EMs [83]. Furthermore, significant amounts of scrap and residues are generated during the fabrication of PMs [84]. However, due to the gap between supply and demand, the necessity for recycling REMs is expected to continuously increase [85].

Overcoming the critical challenges related to recycling REMs requires addressing several technological, economic, and market barriers [86].

The technological barriers are among the most challenging. Recycling REMs from end-of-life products is complicated by the difficulty of separating and purifying these elements from other materials. Additionally, the absence of standardized recycling methods and technologies results in inefficiencies and higher expenses. These barriers could be mitigated by substantial investment in advanced recycling technologies, aiming to develop more efficient and cost-effective methods for extracting REMs from complex waste streams. Establishing standardized recycling protocols is essential to streamline processes and reduce associated costs. Collecting and dismantling end-of-life equipment also presents unique difficulties. RE PMs are used in varying quantities, ranging from a few grams in hard drives and electronic devices to around 1–2 kg in hybrid and electric cars and up to 1–2 tons in wind turbine PM generators [87,88]. These requires specific collection and dismantling methods, further complicated by the presence of hazardous materials that require safe handling and disposal to prevent environmental contamination [89,90]. Developing an integrated supply chain network to facilitate the efficient collection, transportation, and processing of REM-containing waste is thus mandatory [91].

Economic barriers are also significant. The high initial investment needed for recycling facilities and technology development, coupled with fluctuating REM prices, challenges the economic viability of recycling operations [92]. Potential solutions include providing financial incentives and grants to support investment in recycling infrastructure and technology development. Establishing long-term contracts and price stabilization mechanisms can also help mitigate the economic risks associated with price volatility.

By addressing these barriers with targeted measures, the recovery and utilization rates of REMs can be improved significantly, contributing to a more sustainable and resilient supply chain.

#### 4.2.2. Enhancing the Manufacturing Process

Improving the production process of REMs is another viable solution to reduce the amount of newly mined materials. By increasing efficiency through optimizing metal extraction, more REMs can be obtained from the same quantity of mined ore, leading to minimized energy utilization and reduced environmental impact. The production of REMs involves several complex technological stages, including mining, concentration, separation, purification, reduction, and final production. To achieve sustainability, it is crucial to enhance each of these stages, reducing material and energy use while minimizing the ecological consequences of the entire process [34]. Adopting forward-thinking strategies can significantly enhance the sustainability of REM processing [93].

#### 4.2.3. Replacing or Removing Rare Earth Metals from the Products

Another way to decrease the mining of REMs is by substituting or eliminating them from the products. However, when it comes to RE PMs, replacing them can result in substantial performance losses [94]. AlNiCo and ceramic (ferrite) PMs are possible substitutes for the high-energy NdFeB and SmCo RE PMs [95]. Nevertheless, as previously noted, achieving the same magnetic effect as RE-based PMs requires significantly larger quantities of these materials. This results in an increase in the volume and weight of devices that incorporate PMs, which is not suitable for numerous high-demand applications.

Extensive research is underway to develop PMs that do not rely on REMs yet exhibit excellent magnetic properties and meet other critical physical requirements. Below, some of the leading research areas currently advancing in this field are outlined, without claiming to cover all approaches [96]. A prominent area of focus is iron–nitride compounds, particularly Fe_16_N_2_, which show potential for high magnetic performance, including energy densities comparable to those of NdFeB magnets while remaining RE-free. However, significant challenges remain in terms of stability and manufacturability, as these compounds are highly susceptible to oxidation [97].

Manganese has become a focus of research in developing RE-free PMs due to its abundance, low cost, and high magnetic moment potential. Alloys like manganese–aluminum (Mn–Al) and manganese–bismuth (Mn–Bi) are especially promising for certain applications, as they demonstrate high thermal stability, which is critical for EM applications [98]. However, these alloys currently have lower magnetic energy densities than RE-based PMs and exhibit limited mechanical strength. Researchers are exploring alloy composition adjustments and advanced processing techniques to enhance both their energy density and mechanical durability, aiming to make them more viable alternatives in demanding applications [99].

Another promising category of hard magnetic materials is the “1:12 phase” PMs, which have a unique tetragonal crystal structure with a 1:12 stoichiometric ratio, found in certain RE intermetallic compounds. These are represented as RFe_12_, where R denotes a REM, like cerium, samarium, or yttrium, elements that are more abundant and less costly than neodymium. The 1:12 phase materials offer high magnetocrystalline anisotropy, which is essential for achieving strong magnetic properties [100,101].

High Entropy Alloys (HEAs), composed of five or more principal elements in near-equal proportions (such as FeCoNiCuMn or FeCoNiAlCuTi), are emerging as promising candidates for new types of PMs. Their high configurational entropy leads to unique magnetic properties, including significant magnetic anisotropy and high coercivity, with stable performance even at elevated temperatures. Additionally, HEAs offer excellent mechanical properties, such as resistance to wear and corrosion [102].

In addition to non-REM compounds, numerous research teams worldwide are also studying a variety of RE-free hard magnetic materials, with support from advancements in artificial intelligence (AI), which enhances the speed and efficiency of material discovery [103].

Another strategy focuses on reducing the content of critical heavy REMs in PMs. Specifically, the research aims to minimize the use of dysprosium and terbium, elements crucial for thermal stability and high coercivity in NdFeB magnets, by substituting lighter and more abundant REMs, such as cerium and lanthanum, which are also more cost-effective [104]. For example, NdCeFeB alloys are being investigated to produce lower-cost magnets with moderate thermal stability [105].

Scientific advancements provide the enhancement of the manufacturing technique of PMs, thereby reducing the reliance on REMs. Through the optimization of this process, it is possible to decrease the proportion of neodymium and praseodymium in NdFeB PMs from the existing 30% to 26.5%. Furthermore, a substantial reduction of 2.5% in dysprosium content can also be attained [106].

## 5. Possible Electrical Machine Topologies to Diminish the Dependency on Rare Earth Materials

As stated earlier, a significant share of the processed REMs is utilized in the PMs of EMs. Consequently, advancements in this sector can play a vital role in diminishing the need for newly extracted REMs, thereby alleviating the supply uncertainties associated with these crucial resources, as outlined in Section 4.

Also, the European Committee of Manufacturers of Electrical Machines and Power Electronics (CEMEP), fully supports the abovementioned aim in this industry segment in Europe with a market value greater than EUR 22 billion. Their main recommendations concerning the design of EMs are as follows [107]:

- Develop durable and reliable products for a longer lifetime;
- Improve the network of maintenance and repair services (including availability of spare parts);
- Recycle the metals from end-of-life products;
- Increase the share of recycled materials in production and use highly recyclable materials in manufacturing;
- Use materials based on renewable sources in packaging;
- Intensify design digitalization during the EMs’ for optimizing performance and maintainability resulting in reduced waste and increased lifetime.

Consequently, the engineers involved in the development and manufacturing of EMs can greatly diminish the requirement for newly mined and processed REMs. Next, some significant results in this context will be surveyed. The primary emphasis is placed on technical solutions minimizing the volume of PMs in EMs and devising structures that can be easily dismantled for recycling purposes when they reach the end of their lifespan. Also, the possibility of using non-conventional manufacturing methods and non-RE PMs will be considered.

### 5.1. Electrical Machines with a Minimized Quantity of Used Rare Earth Permanent Magnets

In common EM variants, the amount of RE-based PMs can be reduced by optimizing their volume. This strategy not only decreases the size of the magnets but also significantly lowers the overall cost of the optimized EMs, as PMs account for the largest portion of the total price [108,109].

The use of advanced 3D printing technologies offers another potential solution for reducing the amount of RE-based PMs used in EMs. By utilizing this technology, engineers can design complex structures that were previously impossible to manufacture using traditional methods.

#### 5.1.1. Particular Designs Targeting the Minimization of Permanent Magnet Volume

For EM designers, it is imperative to develop devices with maximum efficiency, the smallest possible sizes, and the least costs. These aims cannot be attained without using the most advanced optimization techniques [110,111]. During this important phase of design, the required volume of the PMs can be also minimized, and thus the need for REMs can be reduced, too.

In recent years, optimization techniques, also within the EM domain, have undergone significant advancements [112,113]. The literature contains several papers that showcase valuable optimization success stories utilizing the most cutting-edge methods [114,115]. Next, some of the most interesting achievements will be considered.

As the permanent magnet synchronous machines (PMSMs) have a great diversity of rotor topologies concerning the placement of the PMs on/inside the rotor, optimization techniques were applied to select the best-suited construction variant for each certain application (upon the needed rated power and speed) [116].

**Figure 5:**
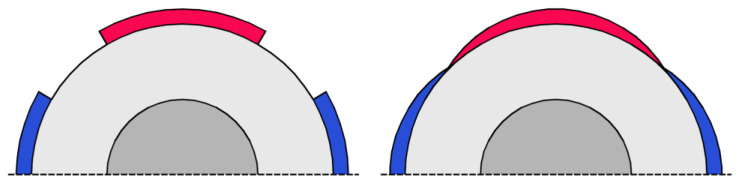
Optimized shape of the surface mount PM in a PMSM.

Some designers optimize the shape of the magnets in PMSMs to minimize their volume and other characteristics. In Ref. [117], the improved PM pole arc was established by using a genetic algorithm (see Figure 5). The upgraded variant of the PMSM preserved the same external dimensions and output power as the original model, yet it accomplished a 15% decrease in PM mass. Additionally, the optimization process led to a reduction in cogging torque and an improvement in efficiency.

By optimally designing the cooling system, the internal temperature of EMs can be lowered [118,119], allowing for the use of NdFeB PMs with lower quality with less dysprosium content [120]. As a result, the quantity of REMs used can also be reduced by this approach.

**Figure 6:**
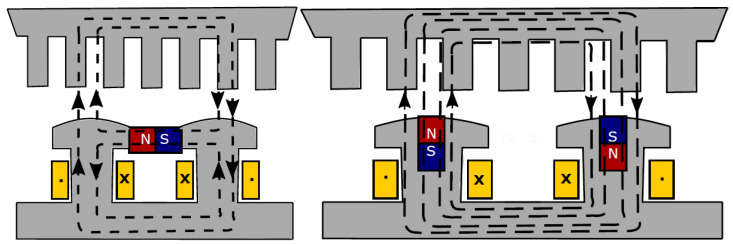
Two variants of hybrid excitation in EMs [121].

Another method to diminish the needed PM volumes in EMs is the utilization of the so-called hybrid excitation. Such machines have both electromagnetic and PM excitation for generating the needed excitation field (as shown in Figure 6); therefore, they require fewer PMs than their fully PM-exited counterparts. Additionally, in the case of such excitation, it is possible to weaken the excitation flux for speed increase, an important requirement in a great variety of applications [121]. Due to the excitation windings, additional heat can be generated within the EM. Furthermore, the increased complexity of hybrid excitation may lead to more demanding maintenance requirements.

In Ref. [122], it is proposed that for a PMSM, the conventional insulator wedges used to secure the coils within the stator slots be replaced with small magnetic components. As a result, the magnetic flux distribution within the EM is enhanced, leading to improved efficiency. This enhancement allows for a reduction in input power, smaller EM sizes, and a lower PM mass, all while maintaining the same output power.

#### 5.1.2. New Manufacturing Approaches

Currently, radical improvements in the field of EMs cannot be performed within the existing manufacturing processes. Hence, a paradigm shift in their development is mandatory. This can be achieved only by applying new approaches to fabrication technologies which could enable the use of innovative topologies and materials [123].

Novel 3D printing technology enabled the creation of all the EM subassemblies layer-by-layer [124,125]. Consequently, iron cores and PM shapes that before could not be manufactured through traditional methods can now be 3D printed, and new perspectives for design engineers to develop innovative EM topologies are opened. By applying such original designs, the needed PM volumes in EMs can be decreased [118,123].

This advanced additive manufacturing technique holds the potential to contribute to a REM economy throughout the PM fabrication process as well. The very precise 3D printing of PMs of practically any shape has the potential to considerably diminish the quantity of lost materials during processing [126]. Furthermore, by using this manufacturing technology, improved control of the PM grain texture can be accomplished, resulting in improved magnetic properties. As a result, the need for adding extra amounts of REMs like dysprosium, terbium, or praseodymium to enhance the properties of the PM alloy can be reduced [127].

While additive manufacturing technologies still face notable drawbacks, such as low printing speeds and high costs, these challenges are gradually diminishing thanks to intensive research focused on improving efficiency, scalability, and process optimization, as well as exploring alternative materials. As these advancements continue, the limitations of 3D printing are expected to reduce, while REM costs rise [128]. Eventually, the balance will favor the general use of 3D printing in the manufacturing of EMs [129]. By then, EM designers must be well prepared for this major technological shift.

### 5.2. Easy-to-Disassemble Electrical Machine Variants

Material recycling from EMs poses a significant challenge due to the need for dismantling and processing the various components made of different materials separately. However, this obstacle can be overcome from the development stage by designing the parts in a way so as to further make the disassembling process easier. For instance, placing the PMs on the rotor in a reachable manner or adopting a modular structure for the armatures can greatly facilitate the dismantling process when these machines reach the end of their lifespan [83,88,107].

#### 5.2.1. Electrical Machines with Easy-to-Extract Permanent Magnets

In recent decades, numerous special EM topologies have been created to substitute traditional ones. Most of them utilize PMs as a source of the magnetic field. Some of these have accessibly placed PMs that can be easily removed from the rotor structure.

**Figure 7:**
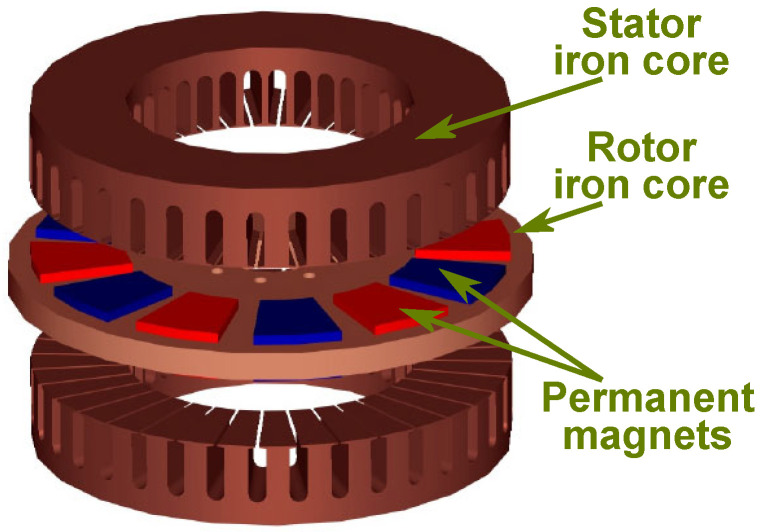
Double-sided axial flux PMSM [134].

Radial flux PMSMs, having the most common construction, are widely used in a great diversity of industries, among them also in renewable energy conversion. They have varied versions considering the placement of the PMs. From a recyclability point of view, the topologies with PMs on the rotor surface are the best, and not those with PMs inside the rotor iron core [130]. The axial flux alternatives of this EM are also used in renewable energy conversion, especially in low-power WTs [131,132,133]. In Figure 7, a compact double-sided axial flux PMSM is depicted [134]. It has two stator armatures and a rotor disk with surface PMs glued on both faces, which can be easily detached during the dismantling of the EM. Such disk-shaped rotor structures can pose mechanical challenges, including maintaining structural integrity and providing robust mechanical support. Additionally, their compact design makes heat dissipation more challenging compared to radial flux motors. Axial flux PMSMs often feature large air gaps and surface-mounted PMs, leading to lower inductances. This makes field weakening more difficult, thus limiting their performance in applications requiring a wide speed range.

PM variable reluctance (VR) EMs are also utilized for these applications, such as flux-switching, flux reversal, and transverse flux variants, among many others [135]. Most of them have topologies making it easy the remove the PMs [136,137].

#### 5.2.2. Modular Electrical Machines

The concept of modularity, which entails breaking down a system into smaller, more manageable components, can also be applied to the design of EMs [138,139]. Both the stator and rotor’s iron cores can be constructed in a modular fashion. This approach not only simplifies the dismantling of EMs but also enhances performance, offering benefits such as greater phase independence and improved fault tolerance. Modular EMs can be fabricated with less material losses and can be more easily repaired. A great diversity of segmental PM EMs has been recently proposed in the literature [140,141]. Nevertheless, for exemplifying this topology approach, only two variants are detailed next.

**Figure 8:**
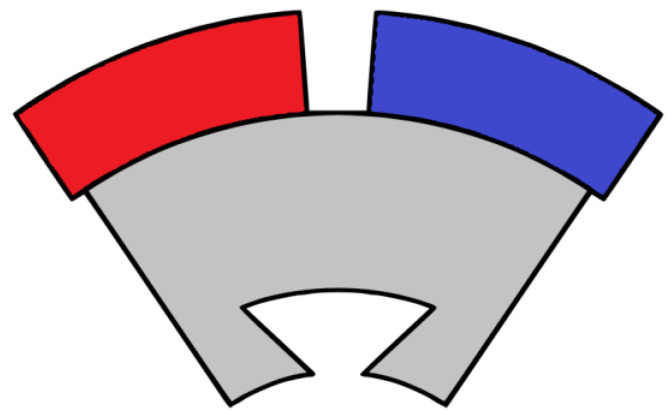
A rotor segment of a modular surface-mounted PMSM.

Figure 8 presents a segment of a modular surface-mounted PMSM rotor, which includes two PMs magnetized in opposite directions, glued on an iron core piece featuring a dovetail slot for secure attachment to the shaft [142]. This design allows for easy disassembly of the rotor and straightforward extraction of the PMs for recycling, similar to the approach outlined in Ref. [143].

**Figure 9:**
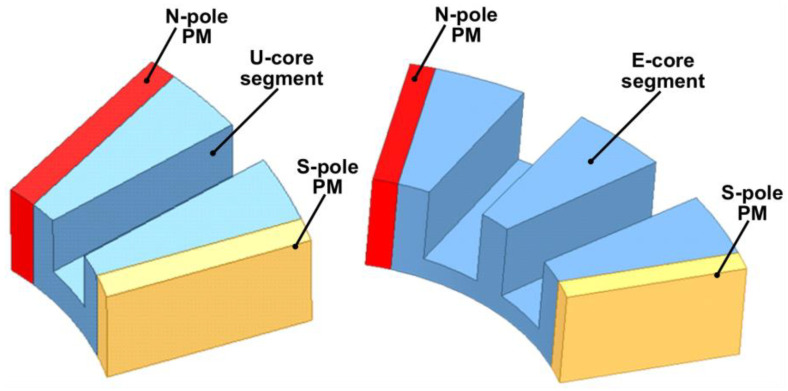
Two types of stator modules of a flux-switching PM machine [144].

Flux-switching PM machines are typically designed with segmented stator iron cores. Two neighbored iron core modules with slots for concentrated coils are separated by a PM (as illustrated in Figure 9). The adjacent PMs are magnetized alternately to optimize the magnetic flux concentration within the passive rotor [144].

Modular EMs have a common drawback: the use of separate iron core segments joined by various additional components can reduce overall structural rigidity [140].

### 5.3. Electrical Machines Using Non-Rare Earth Permanent Magnets

The use of EMs with non-RE PMs holds the promise of reducing the dependence on REMs [145]. In most of the instances documented in the literature, ferrite (ceramic PMs) is suggested as a solution for this objective. To attain the required exigent performance levels of the EMs with these hard magnetic materials, significantly larger volumes of PMs are necessary, as depicted in Figure 1. Consequently, careful design is imperative to prevent EMs from having exaggerated volume and mass.

**Figure 10:**
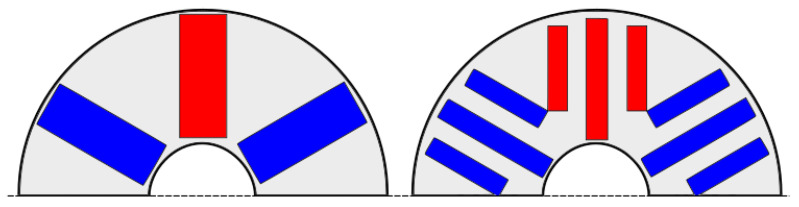
The single and multiple-layer PM arrangements in spoke-type PMSMs.

Placing a larger quantity of PMs within the rotor iron cores is a true design challenge. A potential solution, illustrated in Figure 10, involves replacing a single-spoke-type PM with a multiple-layer structure [146]. This modification allows for a greater volume of PMs to be inserted into the rotor while maintaining its same outside diameter.

**Figure 11:**
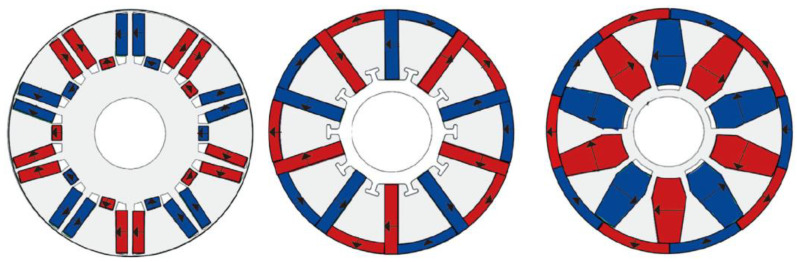
Different placement possibilities of the ferrite inside the rotor of the EMs [147].

Other proposed technical solutions to increase the volume of PMs inside the rotor are given in Figure 11.

Researchers have also suggested the adoption of outer rotor topology, allowing for larger outer diameters in EMs and increased radial direction length for PMs [148]. Innovative suggestions have also been put forward to only partially replace RE PMs with ferrites in EMs. A hybrid PM excitation system, incorporating both NdFeB and ferrite PMs, can be utilized. The two different PMs can be aligned in an extended configuration, positioned as spokes within the rotor of PMSMs [149].

The design and manufacturing processes become more complex when integrating different types of magnets. Achieving optimal performance necessitates the careful tuning of the magnetic circuit to balance the contributions of both types of PMs. This balancing act is also challenging due to the differing magnetic and thermal properties of the magnetic materials. Ensuring precise alignment and optimal placement of the PMs can also pose difficulties. While the use of non-RE PMs may reduce costs, the overall savings could be offset by the increased complexity in design and manufacturing.

There are several already consecrated special EM topologies, such as flux reversal [150], flux-switching [151], or a PM-assisted synchronous reluctance machine (SynRM) [152], which allow for the use of ferrite for excitation without compromising performance [153].

All the previously mentioned EMs are synchronous and can be easily integrated into existing power systems. They can be driven by standard power electronic devices commonly used in industrial applications. Advanced and well-established control strategies, such as field-oriented control and direct torque control, can be applied regardless of the specific construction of the PMSMs. Each of these EMs exhibits high efficiency and adequate torque-speed characteristics, making them suitable for integration into existing power systems without significant modifications.

### 5.4. Electrical Machines Without Rare Earth Permanent Magnets

Finally, it is crucial to take into account the EM alternatives that do not incorporate all PMs in their structures. These alternatives play the most significant role in reducing the demand for REMs.

As was already stated, PM EMs provide a multitude of benefits when compared to alternative topologies. These advantages encompass exceptional efficiency, as well as remarkable power and torque density. Nevertheless, it is worth noting that other types of EMs, which do not incorporate PMs for their excitation, can reach nearly similar performance levels, especially with careful and precise design optimization. Feasible options include induction machines (IMs) and VR machines.

#### 5.4.1. Induction Machines

IMs and PMSMs are engaged in fierce competition within the market [154]. This rivalry extends to the realm of wind energy conversion, where designers face the decision of choosing between the doubly-fed IM and the PMSM for power generation purposes. The intense competition between these EMs catalyzes research efforts, leading to improvements in both variants.

Double-fed IMs offer several benefits when used in WTs. These include the absence of PMs in their topology, the need for a lower grid-side power converter, and reduced initial capital costs. However, it is crucial to acknowledge certain drawbacks. One such issue is the rotor’s complexity, which necessitates contact brushes and rings, resulting in increased maintenance needs. Additionally, these machines have limited direct grid connectivity and exhibit more total expenses throughout their entire life cycle.

The choice between these two EMs poses a genuine challenge. When making this decision, economic factors may carry more weight than technical considerations, as they have a greater impact on the ultimate choice. In this context, the current cost of REM-based PMs is anticipated to have a crucial influence [155].

#### 5.4.2. Variable Reluctance Machines

VR machines, having a particular working principle based on minimizing magnetic reluctance, are newcomers to this market [135]. Although there are numerous topologies cited in the literature, only two variants emerged as significant contenders for PM EMs: the switched reluctance machine (SRM) and the SynRM.

SRMs are the most well-known VR machines. Their main benefits are the simplicity, robustness, low losses, and low price of the passive rotor. SRMs mandatorily require a control system for optimal operation and are prone to torque ripples and noise. They can also be employed in renewable energy conversion, as the absence of self-excitation can be compensated for with suitable control strategies [156].

The SynRM is a highly favored VR EM in the market due to its numerous benefits besides those of the SRMs, such as very high efficiency and compatibility with all-purpose frequency converters. Furthermore, it offers excellent dynamics and requires minimal maintenance [157,158]. SynRMs, like SRMs, also hold great potential in power systems and require a capacitor bank to be connected to the stator windings for the necessary reactive power in the standalone generator mode [159,160].

## 6. Conclusions

Transitioning to a more sustainable future is essential for achieving economic growth that is independent of fossil fuels and the overuse of natural resources. There is a significant demand for eco-friendly solutions, especially in the field of electric power generation, as it contributes significantly to carbon emissions and environmental degradation. By increasing the use of renewable energy resources, the carbon footprint of power systems can be significantly reduced, helping to mitigate the adverse effects of climate change. Hence, there exists an escalating requirement for PM EGs that nearly solely utilize high-energy density RE PMs. However, the increased demand for REMs is encountering significant challenges in terms of availability.

To ensure a reliable supply of these critical resources, governments need to take decisive action by offering tax incentives and enacting supportive legislation that encourages sustainable extraction and innovation. Moreover, there is an urgent need to develop more sustainable management practices for these resources, which can be effectively accomplished through the adoption of circular economy strategies. By promoting the reuse, recycling, and improved utilization of REMs, the global demand for newly extracted resources can be significantly diminished. However, currently, only a limited amount of these metals are obtained from end-of-life devices. Conducting comprehensive research on recycling methods, coupled with the implementation of targeted supportive policies, will undoubtedly contribute to the expansion and long-term economic viability of recycling efforts [161].

Achieving the objective of reducing REM usage requires advancing production technologies and exploring alternative manufacturing methods for REM-containing products, such as EMs. These efforts can also lower both capital and maintenance costs. The responsibility for these improvements lies primarily with research and development engineers.

There are several approaches available to EM designers to minimize the use of RE-based PMs. These comprise reducing or eliminating the amount of these crucial materials. Furthermore, using PMs that do not contain REMs, such as ferrite, presents a viable alternative. To streamline the recycling of PM EMs, designers have proposed easy-to-disassemble configurations that facilitate the efficient removal of the PMs.

Finally, it can be concluded that there are numerous options available to address the supply risks associated with REMs. It is essential to understand that even with all these solutions in place, this hazard cannot be eliminated. Hence, it is crucial for all researchers engaged in this field to persist in their endeavors to minimize this potential risk, as it could surely jeopardize the sustainable future of humanity.
